# Rethinking NGOization as Postfeminist Practice: Interstitial Intimacies and Negotiations of Neoliberal Subjectivity in Violence Prevention

**DOI:** 10.3389/fsoc.2021.654909

**Published:** 2021-05-31

**Authors:** Proshant Chakraborty

**Affiliations:** School of Global Studies, University of Gothenburg, Gothenburg, Sweden

**Keywords:** interstitial intimacy, NGOization, postfeminism, frontline workers, gender-based violence, India, urban poor neighbourhoods

## Abstract

The decade of the 1990s marked the rise of postfeminism, a series of discursive, mediatized and intellectual interventions that furthered, but also broke away from, past forms of feminist theory and practice. This period also witnessed the global proliferation of non-governmental organizations (NGOs) and the “NGOization” of feminism, referring to the cooption and erasure of critical social movements. Beyond their temporal instantiation in the 1990s, postfeminism and NGOization converge and entangle in everyday practices of women’s NGOs and organizations. In this article, I examine such convergences and entanglements as they unfold in an NGO’s community-based program to prevent violence against women and girls in Mumbai’s urban poor neighborhoods. Such programs create new forms of femininity and womanhood among women who participate in interventions as frontline workers. These women navigate complex pressures of communitarian gender norms, disciplinary regimes of professionalization and quantification, and the vicarious harm of supporting survivors. Their affective caring labor, thus, is facilitated by and produces what I describe as *interstitial intimacies*, which problematize and embody key postfeminist claims, while engendering political actions and contestations under neoliberalism.

## Introduction

Over the last 3 decades, the term “postfeminism” has signified theories and practices that have moved away from the radical or critical promise of prior feminist interventions, a turn that has emphasized mediatization, performativity, and identity and agency (Brooks 1997; Gamble 2006; Genz and Brabon 2009). Around the same time, feminist practices and mobilizations, especially on issues like gender-based violence (GBV), have been deeply implicated in regimes of transnational neoliberal governmentality (Merry 2006; Merry 2016). This development—which has unfolded across global and local settings—has most prominently been referred to as the “NGOization” of feminism, or the depoliticization and cooption of critical feminist movements under neoliberalism (Alvarez 2009; Bernal and Grewal 2014a; Roy 2015).

Entanglements between postfeminism and NGOization are not simply temporal; these have produced a global proliferation of actors and institutions, like non-governmental organizations (NGOs), that have emerged as key mediators in feminist contestations (and collaborations) with states under neoliberalism. Such entanglements have led to the expansion and deterritorialization of sites of feminist struggles, and have shifted focus toward embodied, affective and performative aspects of feminist practices. These entanglements have had contradictory or paradoxical effects in contexts such as Global South cities. On the one hand, they have led to women’s increased participation in the burgeoning social sector workforce, but on the other, they have also made their lives more precarious and even deepened other forms of social marginalization and precarity (Baillie Smith and Jenkins 2012; Jakimow 2010; Roy 2019; also, Sangtin Writers and Nagar 2006; Sharma 2006). And insofar as these developments have led to the “NGOization” and depoliticization of feminisms, they also signal newer and unanticipated forms of postfeminist practices, engendering unique forms of politics that exceed the logics of neoliberal subjectivation and reassert commitment to foundational feminist values and ethics, like care, relationality, and critique of patriarchal power (Roy 2011; Bernal and Grewal 2014b; Roy 2017), as well as intimacy, affect, and desire (Wiegman 2010; Roychowdhury 2016; Freeman 2020).

Drawing on ethnographic fieldwork with women frontline workers engaged in an NGO’s violence prevention program in Mumbai’s urban poor neighborhoods, I explore how NGOization constitutes a form of postfeminist practice—particularly in how it unfolds, and is resisted, subverted and repurposed by frontline workers. It is a practice that not only embodies but also moves beyond the apparent contradictions in postfeminist theory and discourse, namely a reassessment of culturally-defined notions of femininity and gender relations. Inasmuch as both postfeminism and NGOization signify depoliticization and individuation (for which they are also critiqued), this article explores how their mutual entanglement signposts newer and unanticipated practices.

I use the heuristic “interstitial intimacy” to refer to such postfeminist NGOized practices and negotiations. Interstitial intimacy points toward ways that feminist activists and agents—women frontline workers, in this case—are implicated in modes of neoliberal governmentality involving NGOs and the Indian state, as well as how they draw on their socially and culturally-inscribed positions of womanhood to resist and subvert such governmentality, and engender forms of collaboration and support. Interstitiality indexes the malleable, open-ended, and overlapping socialities and materialities within which NGOized interventions operate, especially concerning the importance of women and girls’ socially reproductive care work. Intimacy refers to the microsocial and microspatial nature of such socialities, especially in spaces like urban poor neighborhoods or slums—the site were many NGOized interventions are often located. Intimacy also critically evaluates how global and transnational flows of governance shape local encounters between NGOs, local communities, and women. It also challenges the normativity of globalized idioms, concepts and logics which privilege standardized and quantified indicators (Merry 2016).

This article is based on ethnographic research that I conducted with a local NGO that works toward preventing violence against women and girls across urban poor neighborhoods in Mumbai, India. I call this NGO Vinamrata. Vinamrata’s programs emerged in Dharavi, one of the largest urban poor neighborhoods in India and Asia, in the late-1990s—a decade which saw both, the global proliferation of NGOs and the wide-spread adoption of neoliberal and market-driven policies in India’s public healthcare and social sectors (Gupta and Sharma 2006; Rao 2010; Gupta 2012). However, Vinamrata’s role in this framework was somewhat ambivalent. On the one hand, they played an important role in Mumbai’s civic healthcare system precisely as they became part of various state schemes on maternal and child health and nutrition. Their women’s groups augmented the city’s precarious and feminized healthcare infrastructure, while their violence prevention program was recognized as a service provider under India’s Protection of Women from Domestic Violence Act 2005. On the other hand, much of Vinamrata’s initiatives, which were led by women in both positions of leadership and on the ground, were a form of feminized intervention into the state’s bureaucratic-rational practices.

Thus, Vinamrata’s interventions foregrounded wellbeing, reciprocity, and care—responsibilities that are disproportionately borne by women as a form of socially reproductive labor. As I have argued elsewhere, frontline workers appraise such mobilizations of care in critiquing violence and gendered inequality in their communities (Chakraborty 2021a), and at times even question how NGOs are embedded in state and neoliberal structures that privilege abstraction and quantification over connection and concern (Chakraborty 2021b). In other words, we can perhaps frame Vinamrata’s work as situated at the interstices of neoliberal governmentality, feminist social work, and the state’s bureaucratic-rational practices. In such conditions, I use the heuristic interstitial intimacy to foreground frontline workers’ everyday negotiations that draw on, push against, and rework these overlapping and at-times contradictory logics.

I devoted a substantial part of my fieldwork with Vinamrata’s women frontline workers. These frontline workers include both, staff members who are paid employees, and volunteers who are known as “sakhis” (the feminine word in Hindi for “friend”). Frontline workers live and work in the very communities and neighborhoods—or *bastis* as they are colloquially known—where Vinamrata’s programs are based. They identify and support survivors of violence in their neighborhoods and communities, work with Vinamrata’s crisis counsellors and police stations, and engage in local politics of urban commons^1^.

As they are situated within such postfeminist and NGOized convergences and entanglements, frontline workers’ everyday practices are predicated on the self-fashioning and assertion of gendered and feminized logics of care and intimacies, which also frame patriarchy and patriarchal power as deeply entrenched and unequal social structures that produce conditions of violence (Ortner 2014; also, Chakraborty 2021a). Insofar as they draw on the language of care, feminine values, and heterosexual conjugality and domesticity, frontline workers navigate the complex divisions between femininity and feminism (Mahadevan 2014). I focus on NGOization as a particular form of postfeminist practice, and examine how women’s NGOs both re-signify and engender newer forms of femininity and womanhood. My ethnographic materials show how frontline workers navigate complex pressures of communitarian gender norms, disciplinary regimes of professionalization and quantification (Merry 2016; Roychowdhury 2016; Roy 2019), and the vicarious harm of supporting survivors (Haldane 2017).

The rest of this article is structured as follows. In the next section, I provide a historical and conceptual background to postfeminism and NGOization, particularly their convergences and entanglements. I then provide a brief overview of the heuristic interstitial intimacy, and then discuss my ethnographic fieldwork with Vinamrata’s frontline workers. Following this, I present four ethnographic accounts. The first two are drawn from my work with sakhis, Vinamrata’s voluntary frontline workers. These accounts describe how two groups of sakhis, living in two different neighborhoods, presented expressedly divergent perspectives on their gendered and feminized social locations and dispositions. Next, I synthesize these accounts to further develop the idea of interstitial intimacy, stressing how it reflects convergences and divergences in NGOized interventions, and attends to wider social, material and historical processes.

The two ethnographic accounts that follow are drawn from my work with Vinamrata’s community workers who, like the sakhis, are also urban poor women, but are part of an increasingly professionalized workforce. In particular, these accounts describe pressures of quantification and professionalization that these women deal with. I show that, despite Vinamrata’s intervention program incorporating diverse institutional and community actors, the underlying logics of NGOization produce further divergences in their instantiations of interstitial intimacy. Yet, having shown how postfeminism and NGOization converge and entangle, I argue that frontline intervention work is deeply inflected with care, which materializes as a form of emotional and caring labor. In so doing, frontline workers’ interventions go beyond restrictive critiques of NGOization; instead, they assert the importance of the semantic instability between languages of “projects” and “movements,” and underscore the importance of adopting hybrid forms of engagement that go beyond conventional political strategies.

## Postfeminism and NGOization: Convergences and Entanglements

While hard to define, scholars generally agree that postfeminism emerged in the late-1980s and 1990s as a departure from past forms of radical and socialist feminisms of the 1960s and 1970s. This departure, in part, has been understood as a “backlash” against feminist politics, as well as a generational divide between older and younger feminists (Brooks 1997; Gamble 2006; Genz and Brabon 2009; Ortner 2014). Postfeminist representations and articulations are manifest across mass mediatized forms of feminine expression that assert selfhood, autonomy, choice, and independence (Gamble 2006). Thus, postfeminism is a contested term, imbricated as it is in politics of liberation, choice and agency, on the one hand, and discussions over cooption and depoliticization, on the other (Brooks 1997; Gamble 2006; Ortner 2014).

As Genz and Brabon (2009) discuss in their volume on “postfeminism,” the term, among other things, has been understood as signaling either a radical break and departure from past iterations of feminist theories and movements—a “genealogy that entails revision or strong family resemblance,” or a “precarious middle ground typified by a contradictory dependence on and independence from (past forms of feminism).” Cautioning against any original or authentic definition, they instead locate postfeminism’s emergence in complex and overlapping public domains at the “intersections and hybridization of mainstream media, consumer culture, neo-liberal politics, postmodern theory and, significantly, feminism” (4–5). Similarly, Gamble (2006) traces its origins in 1980s United States and North American mass media, paying particular attention to blurred boundaries between how postfeminism was represented, and what it entailed as a form of critical practice and discourse. Crucially, Gamble differentiates between “postfeminism” and “third wave feminism”—terms that are often used interchangeably. She draws on rich theoretical and activist interventions, particularly from Black and Third World women scholars (such as bell hooks), to show third wave feminism has links with political activism, and is “more than just a theory, but an approach that will actively work against the social injustices which still form part of the everyday experience of many women” (43–44).

In contrast, Brooks (1997) views postfeminism as a critical theoretical movement that aligns with other anti-foundationalist movements, such as post-modernism, post-structuralism, and post-colonialism, especially as it challenged dominant understandings of structure, agency, and epistemology (34). She further differentiates popular forms of “post-feminism” in mass media discourses from the analytic and political project of postfeminism, which is crucially “not a depoliticization of feminism but a political shift in feminism’s conceptual and theoretical agenda,” and “represents a dynamic movement capable of challenging modernist, patriarchal and imperialist frameworks” (4). Similarly, Hall and Rodriguez (2003) use public opinion data in the United States to examine public support for postfeminist perspectives. They identify four postfeminist claims: 1) overall support for the women’s movement has dramatically eroded 2) because some women are antifeminist, and 3) believe that the movement is irrelevant, and 4) have adopted a “no, but … ” version of feminism. Their analysis of public opinion data throughout the 1990s finds little support for these claims, and instead showed that not only has support from the women’s movement “increased or remained stable” (888) but that women—and particularly younger and African American women—supported women’s movements and saw it as beneficial (891–96).

While these scholarly interventions are generally situated in the North American cultural and political context, Sherry Ortner (2014) further explores the global genealogies of feminism and postfeminism. She observes that feminist movements and theories—especially as they emerged in Global North institutions—had been challenged by postcolonial feminist, political and anthropological scholarship. For instance, Mohanty (1988) critiques Western feminist scholarship for discursively producing the singular and reified representation of the “Third World Woman,” which erases the complicity of Western feminism in discourses and practices of colonialism. Similarly, anthropologists like Ong (1988), Mahmood (2001) and Abu-Lughod (2002) critique the ways in which non-Western women are often configured as the “other” of Western feminism, and are seen as lacking agency, saturated with cultural difference, and in need of rescue—which thus legitimate neo-colonial forms of violence. In particular, Ortner (2014) draws attention to how critical questions on the evolving and changing nature of “patriarchy” under neoliberalism have somewhat escaped both, postfeminist discussions and discussions on/around postfeminism. She argues that patriarchy and patriarchal power are manifest in subtle, changing ways in intimate spaces and encounters, whilst being intertwined with other structures of power, like colonialism, capitalism, imperialism, and so forth (533–34).

Around the time that debates on postfeminism emerged in the Global North, issues like gender justice, women’s rights, and prevention of gender-based violence—especially in the Global South—were steadily becoming a global agenda in the decades between the 1970s and 1990s. During this time, the United Nations’ World Conferences on Women culminated with the Beijing Conference in 1995, which widely recognized the importance and legitimacy of institutions like non-government organizations (NGOs) in global gender justice movements (Merry 2006). Yet, such developments signified complex macro- and micro-social and political shifts, where institutional and mediatized interventions transformed feminism from a political movement to a programmatic approach that could address issues of gender inequality and gender-based violence. Importantly, the 1990s also marked the apogee of global neoliberal governmentality and substantial restructuring of state power and capacities in countries like India (Gupta and Sharma 2006; Sharma 2006; Gupta 2012; Roy 2015). In such contexts, NGOs became part of the state’s social welfare infrastructures, but were also responsive to, and drove, market- and global institution-led interventions. This is what critical scholars have described as the “NGOization” of feminism and women’s movements (Alvarez 2009; Bernal and Grewal 2014a).

Like postfeminism, NGOization too is a contested term. While some critics like Fraser (2009) have argued that the ideals of second-wave feminism converged with modes of neoliberal governmentality in the 1990s, others have made more nuanced assessments, particularly regarding the co-option of feminism (Roy 2015, 2017; also, Eschle and Maiguashca 2018; Wiegman 2010). For instance, Roy and Grewal’s discussion on co-option also focuses on the generational divide between “older” and “younger” generations of feminists, and underscores the inherently pluralistic—though at times fractured—nature of feminism and feminist activism (Roy 2017). Whereas others like Eschle and Maiguashca (2018) have found that contemporary feminist discussions tend to frame co-option and resistance as dichotomous terms. They build on critiques that point to the limited ways in which both feminism and neoliberalism are understood, and indeed how these critiques exert disciplinary power on feminists themselves. Wiegman (2010) also critiques the charges of co-option, arguing instead that feminism is a social movement and theory that is continually reinterpreted and reused in struggles precisely because it is motivated by a desire to fulfill its political potentials.

Thus, the convergence and entanglement between postfeminism and NGOization not only underscore how feminist practices and theories have transformed over the last 30 years, but also point toward emergent modes of practice that unsettle prior assumptions as well as engender new forms of engagement. From this vantage point, it is necessary to both historicize neoliberalism, which is itself an unruly and ambivalent concept (Kingfisher and Maskovsky 2008), and foreground how it acquires salience in the everyday workings of NGOized interventions. For one, as Chhabria (2019) has recently shown, capitalism and neoliberalism have profoundly disruptive histories in postcolonial cities like Mumbai, where these have shaped the very fabric of urban space and subjectivity—especially in areas that become classified as “slums.”

Still, as Chant and McIlwaine (2016) have shown, women and girls’ socially reproductive care in cities remains both central and marginalized under contemporary neoliberal conditions as it was under industrial modes of capitalism. In such conditions, the political economy of women’s NGOs and organizations were an extension of feminist practice emerging from women’s movements, which nevertheless became complicit in neoliberal state and market-driven power (Roy 2015; Roy 2017). As mentioned above, the former included NGOs becoming proxies or providers of state services, whereas the latter included philanthropic funding, donations, and corporate social responsibility programs—indeed, both trajectories have framed Vinamrata’s work in the last 2 decades.

Critical feminist and anthropological research, however, have problematized these trajectories by exploring how local NGO worlds and practices are marked by complexity and diversity (Roy 2017). While others have shown how local communities resist feminist and NGOized interventions, often to the detriment of survivors of violence (Datta 2012), collaborative research among scholars and activists have drawn on reflexive methods to problematize both, the intersections of caste, class, gender, religion, and sociospatial location, and the hierarchies of donor-driven women’s empowerment (Sangtin Writers and Nagar 2006). My previous writings on Vinamrata’s frontline workers have contributed to this discussion by showing how their work involves intersections of care and critique wherein they mobilize their socially inscribed role as carers to critique gendered inequalities and forms of violence (Chakraborty 2021a). Their affective and embodied labor aligns the processes and outcomes of programs with the lived realities and experiences of communities and overcome disjunctures produced by urban and NGOized precarity (Chakraborty 2021b).

## Interstitial Intimacies

I use the heuristic “interstitial intimacies” to refer to how frontline workers are, at once, part of complex, overlapping social structures, and how they draw on these multiple, refracted social identities and logics to negotiate violence. As I have discussed elsewhere, frontline workers’ performance of care is a form of interstitial labor which aligns Vinamrata’s organizational processes and outcomes with the needs and expectations of communities, and overcomes disjunctures in the form of precarity produced by pressures of professionalization and sociomaterial vulnerability in urban poor neighborhoods (Chakraborty 2021b). My use of interstitial intimacy in this article, however, is more grounded, in that it reflects and corresponds to how frontline workers conceptualize their relationships with each other, and with their families and communities, through a notion of intimacy that articulates and engenders collective desires and affects.

My use of “intimacy” draws on feminist and anthropological discussions that move away from reducing intimacy to relations of sexuality and conjugality, and instead focuses on how globalization both shapes existing, and produces new, intimate spaces and encounters (Sehlikoglu and Zengin 2015), and how intimacy brings together and crosses the lines between private and public spheres and relations (Wilson 2012). Feminist theorists and anthropologists have also situated intimacy within historical and cultural frameworks that shape desire, affect, and emotions, while also recognizing these as deeply political questions (Ahmed 2004; Wiegman 2010; Freeman 2020).

At the same time, my use of the word “interstitial” is inspired by urban studies and the anthropology of emotion and affect. In urban spaces, interstices go beyond dichotomous social and material formations, like core–periphery or center–margin. In his introduction to an edited volume on urban interstices, Brighenti (2013, xvi–vii) argues against the dominant tendency of viewing interstices as empty or gaps; instead, interstices are an active component of the urban fabric, emerging through complex processes of urbanization. My use of interstitial intimacy also draws on the work of Sara Ahmed (2004). Ahmed argues that emotions do not reside in subjects or objects, but “align individuals with communities—or bodily space with social space—through the very intensity of their attachments,” binding subjects together to create the effect of a collective or “coherence” (119). In this way, interstices correspond to how Wies and Haldane (2011, 1) draw on Sally Merry’s work to define front-lines as “small spaces of interactions”—which I take to mean conditions of intensification, density, and congealing of social relations (Simone 2004).

Interstices, however, are not simply in-between spaces; nor are frontline workers simply in-between or liminal actors (Turner 1991), shifting between foregrounds and backgrounds of their social worlds as it were (Goffman 1956). Instead, interstitial intimacy signifies an intricate reworking of frontline workers’ socially-inscribed and feminized roles as carers and their neoliberal subjectivation as a class of precarious workers; it reflects both, the proliferation of NGOs and NGOized programs, and how these challenge, rework and reconstitute everyday intimate relationships and values in urban poor neighborhoods—from conjugal, neighborly and communitarian relationships, to relationships between urban inhabitants and local and global institutions, like states, police, courts, and funding agencies. In other words, as the following ethnographic accounts would show, interstitial intimacies refer to how women frontline workers’ draw on their multiple, refracted identities in everyday interventions to prevent violence: they are wives and mothers; but also friends and neighbors, as well as activists and professionals—often simultaneously and in ways that these identities are inseparable yet distinct.

## Ethnographic Groundings

Between 2014 and 2019, I conducted over 15 months of ethnographic fieldwork with Vinamrata’s frontline workers. During this time, I was associated with their violence prevention program in Dharavi, one of the largest urban poor neighborhoods India and Mumbai. I collaborated with them as an independent research consultant and applied anthropologist, conducting several formative and evaluative studies on their frontline workers and various community groups.

My fieldwork encounters with Vinamrata’s program were deeply informed by my training in and commitment to ethnographic research, where the participant-observation ethos often entailed following the rhythms of everyday life. As a field-based research method and genre of writing and analysis, ethnography relies on sustained participant-observation with social groups and is used alongside *contextual* and *comparative* analysis to generate anthropological theories (Sanjek 2014). Among other things, this meant that apart from the time I spent in meetings and discussions with my researcher colleagues at Vinamrata’s main offices, I tried to mirror my everyday work routines during fieldwork to that of my colleagues and collaborators in the violence prevention program’s community team.

My fieldwork experiences also raise important ethical issues of conducting ethnographic research while being embedded in organizational settings. Vinamrata’s frontline workers were, at once, my colleagues and research participants. My interactions with them required a complex form of “impression management” (Berreman 2007), where I had to navigate between—but also creatively integrate—my roles as a consultant and ethnographer. For instance, as an ethnographer I spent time with my colleagues and collaborators, participating in everyday interactions beyond “data collection,” like shared lunches, trainings, meetings, and long walks between the community center and various bastis in Dharavi. The conversations and interactions that took place in these moments profoundly influenced my understanding of frontline work and shaped my ability to collect relevant evidence through interviews and focus-group discussions.

While collecting such data, my researcher colleagues and I would use standardized protocols during interviews and FGDs to ensure we followed the ethical principles of informed consent, voluntary participation, and confidentiality. Yet, in my everyday ethnographic encounters, I had to make my participants conscious of the fact that I would like to collect notes about particular interactions; obtaining consent thus involved conversations and discussions regarding the importance of these seemingly mundane interactions, where I often impressed upon my participants why and how their experiences and insights were relevant to our research. This also included crucial conversations regarding which parts of our interactions we did not record, such as critical comments or personal stories.

My fieldwork interactions also involved negotiating my privileged position as an upper-caste, upper-class *cis*-gendered man in ways that did not foreclose the possibility of doing “cross-sex” research (Gregory 1984; Berliner 2008; Thomas 2017). For instance, in interactions with basti women and sakhis, I would take more of a passive role—being reflexively aware of my privileged position as a man in a space that was feminized. In such instances, I would inhabit the role of someone who “belonged” with the organization, whilst also making my “outsider” identity more apparent by visibly displaying markers like my backpack and notebook. This also underscored my role as someone whose work was often about documenting evidence which would ultimately be useful to “show” their work (conversely, this also meant that I knew when to not use my notebook). In such cases, I took down fieldnotes as freely and as often as I could, usually to document meetings or group sessions with community workers or group members. Most quotes or exchanges were taken verbatim in the original language (usually Hindi or Marathi), in order to document the exact and precise words and phrases participants used. I also made distinctions between directly observed exchanges and my immediate reflections, which I often scribbled on the margins. I coded my fieldnotes and interview transcripts manually (i.e., without the use of qualitative data analysis software) to generate empirical themes and rubrics (e.g., interstitial intimacy) which were further conceptualized and streamlined while writing up ethnographic accounts.

This model of fieldwork gave social legitimacy to my presence in spaces where strangers, and especially strange and unfamiliar men, are not usually welcome—and, in many cases, for good reason^2^. As part of conventional ethical responsibilities, then, I have assigned pseudonyms to my colleagues and respondents; and in sections where I discuss my collaborative work with Vinamrata colleagues I emphasize so by shifting from “I” to “we.” However, given how experiences and biographies of many community workers’ and sakhis’ are common knowledge within Vinamrata’s organization context and even across several communities in the basti, the ethical principles of confidentiality may not be entirely successful in anonymizing them in the text. Many such stories circulate in meetings and conversations, and are also important pedagogical moments for the team. Despite this, there were numerous conversations that were critical and sensitive which I have not included in the text because my participants requested so. I nevertheless carry these insights as part of my ethnographic knowledge and their concerns and critiques certainly inform my writings and arguments, as well.

## On the Frontlines of Violence Prevention: Ethnographic Accounts

### Affective Encounters, Intimate Collectives

Niharika held up a poster that she and her group had drawn on a chartpaper moments ago as part of a training workshop, while her companions recounted how they experienced *badlaav* (change) over the years they were involved with Vinamrata. One woman described herself as a “*saheli*” (friend), whereas another said she felt like a “social worker.” Another woman added, “First, I would think about only my family, but now there has been *sudhar* (improvement).” She continued, “Earlier, life was boring, but now it is *haribhari* (colorful)!”

Niharika was a sakhi with Vinamrata’s violence prevention program, one among the 150 voluntary frontline workers that they had trained over the years. The audience, largely comprised of other sakhis like her, cheered on encouragingly as they awaited their turn to present posters they had made. We were all seated in small groups on the floor of an auditorium at the municipal hospital in Dharavi, where Vinamrata’s offices and crisis counseling centers were located. This was in July 2015. Niharika, the other sakhis and Vinamrata’s community workers all lived and worked in Dharavi’s various bastis.

At this particular moment, the pace of the workshop had somewhat quietened and taken a contemplative turn, as the sakhis were asked to present their individual and collective experiences of change. The workshop began with about an hour of physical activities and games, something that these sakhis, as middle-aged women living in bastis, had hardly ever engaged with in decades. While nervous at first, the sakhis had enthusiastically come forward to play games like *phugri* (where they held hands and spun around in a circle) and *langdi* (a game of tag, but played whilst hopping on one leg).

When Niharika and her group concluded their presented, she narrated her own story of change, and how she had managed to educate her daughters with Vinamrata’s help. She thanked them and the community workers, but used the plural pronoun “*hum*” (“we/us”). At this point, the trainer who was facilitating the workshop, softly interrupted Niharika. “Why are you saying *hum*?” she asked, and then instructed Niharika and the other sakhis, “Don’t say *hum* … say *mujhe*!” *Mujhe* being the singular pronoun, “I”—which I felt was her way of encouraging the sakhis to assert their individuality. Niharika smiled and awkwardly agreed, after which the other groups took their turn to present their posters.

Neerja said that before she was a sakhi she lived a “half *zindagi”* (half a life), as though she was “living in another’s hand” (*kisi aur ke haath mein*). But now she said that she could “give *nyay”* (justice) to others,” and had her rights and happiness. Leela characterized her transformation thusly, “At first I was a *murgi* (chicken), but now I am a *tota* (parrot)!” Surbhi spoke of how she now possessed *himmat aur hausla* (courage and resolve) to intervene in conflicts by being *bindaas* (without any inhibitions).

**FIGURE 1:**
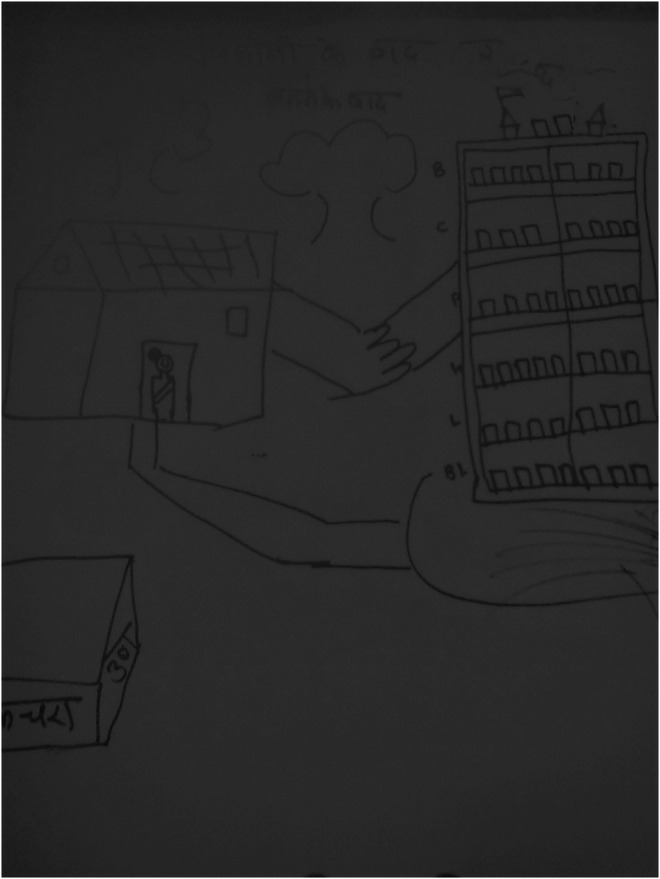
Noori’s poster which shows the organization’s hand meeting with the community. Photograph by author. Note: the photograph has been altered to preserve participant confidentiality.

Finally, Noori, a transwoman sakhi, spoke. She had not known about Vinamrata at first but said that she was here today after sakhis and community workers told her about the program and the work they did. “Abuse against women is wrong,” she continued, and having witnessed incidents like domestic violence and burnings, she “had to do something.” She then presented her poster (Figure 1). A building-like structure stood on one side, and a house on the other. A hand extended from the building and met another extended hand from the house in the middle. She explained, “This is the *sanstha’s* (organization’s) hand reaching out to our homes.”

### Reasserting Domestic Intimacies

In December 2018, I was at one of Vinamrata’s field offices in Shivaji Nagar, a large basti located in Mumbai’s M-East ward in the eastern suburbs. Vinamrata’s violence prevention program has been active in this area for the last 4 years. Their office was a small mezzanine room located in the premises of the local police station, which served as both a crisis counseling and community center. I had tagged along with some colleagues who were about to conduct a training workshop with a group of sakhis. At the time, I was developing training material for Vinamrata’s work with community men who were part of their intervention as allies. Having heard about this group of sakhis, who had been involved in several community actions, I wanted to conduct a short workshop with them to gain insights to incorporate into their work with male allies.

About eight sakhis, some with small children, were seated on the floor of the mezzanine room, with some Vinamrata project workers. After their training concluded, I joined them on the floor, and greeted them—some of whom I had met in a previous event in this neighborhood. The purpose of my workshop was to discuss their vision of an “ideal community” (*aadarsh samuday*), which we could then use to engender accountability among male allies^3^. And having heard about the exemplary work many of these sakhis had engaged in, I was hoping to hear their critical insights regarding the nature of patriarchy, resistance, and solidarity.

To my surprise, however, the discussion veered away from how I had anticipated it would unfold. Instead, what I heard from these sakhis was their version of an ideal conjugality. From the very beginning, the sakhis were hesitant at assigning blame—or even responsibility—of gender inequity and violence to men in their community. One sakhi tried to establish some sort of parity between women and men. “Men are concerned (for their wives) when we are ill (*bimaar*),” she reasoned. For her, this underscored that men do engage in care work, even if under constrained situations. Troublingly, another sakhi added, “Men should see women as (their) sisters or mother.”

A young sakhi, who had attended the workshop with her son, intervened. She emphasized “*soch*”—thoughts, ideas or beliefs—as a particular problem. She reasoned that it was men’s *soch*, as well as that of the entire neighborhood, that was at fault. “This is the source of conflict and jealousy,” she said. She then referred to the notion of an “ideal” community that I had mentioned and said that this vision was not possible in their community, as residents were always concerned about the affairs of others. This social structure, which was also interpolated into the metaphor of *soch*, was something experienced sakhis like her were entangled and complicit in. She referred to it as “*humari soch*”—“our beliefs.”

After a brief minute of silence, an elderly Muslim sakhi spoke. “Men and women are equal,” she asserted, but then added that as women—and wives, at that—“We have to talk to (our) husbands respectfully (*izzat se*) … (They) come home after a long day at work.” She then critiqued the young men in the neighborhood, many of whom are involved in petty crimes and hooliganism (*dadagiri*). Rather than resisting or challenging them, she said, “We should speak nicely to them … refer to them as *aap*,” a pronoun signifying rank or respect. “Then other men and boys would [realize] … they will tell others, “Speak to them with respect!” Another Hindu, middle-aged sakhi, agreed with this, and said that they have some faith in younger men in their neighborhood—many of whom participated in Vinamrata’s program. She reasoned that they have the potential to change, even more so than older men who appeared to have been set about in their ways.

### Interstitial Intimacies, Intimate Affects: Discussion

These ethnographic accounts appeared to be diametrically opposed in their emotional and political orientation. The first workshop was marked by a sense of conviviality, exuberance, and critical reflection. These sakhis emphasized the change and transformation they experienced in being part of Vinamrata, and the power of building relationships—with each other and with the organization. As my Vinamrata colleagues and I have argued elsewhere, such affective encounters between NGOs, community workers, and community women engender change and transformation, which are further facilitated through reciprocal relationships (Chakraborty et al. 2017). The Shivaji Nagar sakhis, in contrast, appeared to look inward, *within* domestic spaces and conjugal relationships, and asserted a view that ostensibly sought to preserve the patriarchal status quo. While some of them pointed out the lack of social cohesion and marginalization in their neighborhood, others reasserted some amount of faith in them, especially young men.

In a crucial sense, these divergent accounts exemplify certain postfeminist contradictions discussed above, namely the continuities and discontinuities between modes of feminist and feminized solidarities and changing matrices of heterosexual and, in this case, conjugal relationships. One could assume—as I initially did at the moment—that the latter sakhis’ beliefs were a form of “benevolent sexism.” According to Glick and Fisk (1997, 121), benevolent sexism is opposed to more hostile forms of sexism, as it “relies on kinder and gentler justifications of male dominance and prescribed gender roles; it recognizes men’s dependence on women (i.e., women’s dyadic power) and embraces a romanticized view of sexual relationships with women.” At the same time, these sakhis’ strategic emphasis on maintaining conjugal relations could also be viewed as what Kandiyoti (1988, 285) termed as “patriarchal bargain,” which refer to strategies adopted by women in male-dominated societies to submit to patriarchal norms in exchange for security and material wellbeing.

Yet, upon further reflection and conversation with Vinamrata’s community workers over the years, I do not think benevolent sexism or patriarchal bargain explain these sakhis’ interventions. Despite the unanticipated turn of our encounter, the community workers and sakhis who lived and worked in Shivaji Nagar had extensively documented multiple interventions they had done to prevent violence, support survivors, and even involve local elected officials and the police. Furthermore, as we saw in the ethnographic account, these sakhis spoke about the wider socioeconomic conditions of precarity and poverty, a mode of urban subjecthood which collapsed distinctions between social, material, and cognitive structures, exemplified in their deployment of the notion of *soch*.

Following Bourdieu (1990, 53), we can interpret *soch* as not just cognitive structures or beliefs, but as a form of *habitus*, that is, “systems of durable, transposable dispositions,” which are structured by and generate the “practical world.” Bourdieu’s rendering of *habitus* signifies material and representational structures (which, in his words, are “objective”) and forms of social action available to actors, who nevertheless have the potential to act in ways that subvert these schemas. Moreover, given the prevalence of gendered violence and the sakhis’ everyday forms of actions to prevent violence, it is also important to draw attention to how gendered subjectivities and social structures are entangled in such Bourdieuian dispositions, what he referred to as “symbolic violence.”

Symbolic violence is the “*violence which is exercised upon a social agent with his or her complicity*” (Bourdieu and Wacquant 2004, 272, emphasis in original). Symbolic violence is a form of *misrecognition*, an inability to perceive violence as violence. It draws attention to both, the social constructivist aspect of gender (the worldview of two biological sexes), as well as how this itself is inculcated and embodied (273). The practices of institutions and individuals, to a great extent, naturalize and embody such violence. Contrary to its semantic formulation, symbolic violence “is exerted not in the pure logic of knowing consciousness but through the schemes of perception, appreciation and action that are constitutive of habitus” (Bourdieu 2004, 339–40). According to Bourdieu, symbolic violence can only be resisted through embodied means that bring about “a radical transformation of the social conditions of production of the dispositions,” rather than purely discursive acts (342).

The affective, embodied, and convivial nature of the workshop in Dharavi was, in many ways, exemplary of the way that Vinamrata’s interventions engaged frontline workers (Chakraborty 2021a). Crucially, this account illustrated how their interventions produced a form of intimacy within the space of the workshop, but one that also transcended it. This was evinced in both, how Niharika referred to herself with the plural pronoun “*hum*” (for which she was corrected by the facilitator, showing how at times even NGO representatives overlook the subtle effects of their work), and how Noori’s visual representation quite literally signified the intimate act of holding hands. In contrast, while the Shivaji Nagar sakhis’ interventions illustrate the problem of how such intimacies are imbricated in precarious lifeworlds, their allusions to and mobilizations of a different register of intimacy—marital conjugality—showed how NGO interventions nevertheless inflect these intimacies with critical notions of equality (e.g., “Men and women are equal”). At the same time, they also refracted domestic intimacy, along with attendant notions of mutual respect and responsibility, onto the public as a means of holding their community members (especially young men) accountable.

These two divergent but related forms of intimacy become interstitial precisely as they foreground connection; not only of drawing closer, but also pushing against. For one, both accounts underscore the material basis of urban poor women’s centrality in managing—and transcending—public and private dichotomies (Chakraborty 2021a). Indeed, as Roy (2003) shows in her ethnography with women in Calcutta’s urban poor neighborhoods, “domestic”—and public—spaces are inherently unstable social formations that develop from particular histories of migration and political economy. She terms this organization of private, intimate spaces as *domestication*, “a polyvalent, organizing concept to detail the logic of double gendering,” which “operates within fields of power” (86). This process of domestication entails a feminization of poverty, where women’s work and wages are undervalued, and they are further discursively placed in a bounded home (which pivots on the public–private binary). In her ethnography, the discourses of working women disrupt these domestic imperatives, and thus, she argues domesticity is something that is negotiated “inside and outside the domus” (87). These negotiations, furthermore, undermine the “false binaries of work and household, production and social reproduction, public and private” (88).

Yet, when urban poor women are part of interventions which explicitly foreground and mobilize affective modes of engagement, we see how negotiations of domestication also articulate desires of conviviality (among sakhis themselves), as well as intimacy and conjugality (in their relationships). This articulation of intimacy counters how urban scholars like Datta (2016) have framed women’s “right to intimacy” in contexts of intimate and domestic violence, where survivors reject outside interventions and instead “absorb” violence that sometimes leads to harm, injury, or even death (Datta 2012). And neither do these desires of intimacy reduce sakhis’ sense of empowerment or agency as purely mental or cognitive, which we see in Roychowdhury’s (2016) work with NGOs that transform women’s mind-sets or save their souls instead of providing material support.

Evidently, this is not the case with sakhis in either of the ethnographic accounts. For one, their interstitial intimacy problematizes insider-outsider dichotomies, as they try helping survivors by being both, caring friends and neighbors and also frontline workers—which we saw in the first account. For another, both groups of sakhis were cognizant of the respective collectives they were part of. For instance, Niharika’s instinctive use of “*hum*” instead of “*mujhe*”—for which she was gently corrected by the workshop coordinator—reflected how the singularity of her experience was rather part of multiple, collective trajectories of other sakhis like her, who were all discussing and celebrating the entanglements of personal and collective experiences of change (*badlaav*). Similarly, the Shivaji Nagar sakhis’ emphasis on both, their conjugal and wider communitarian relationships, underscored the inherently social and suffused nature of their positionality as frontline workers. In order to work efficaciously to prevent violence—which we saw in their well-documented past actions—they re-signified the desire of intimate conjugality in opposition to erosion of social and communitarian respect, expressed in the notion of *soch* discussed above. Even as domestic intimacies were inflected with notions of equality, these were consciously framed and deployed to foreground (and possibly critique) their complicity in these structures (*humari soch*). This also indexed the collectives they were part of, albeit marked by specific communitarian experiences of marginalization,^4^ even as these affective intimacies expressed optimism and possibilities of change (Freeman 2020, 84–85).

### Fractured Intimacies, Navigating Boundaries

On a humid and cloudy July morning in 2015, I arrived at Vinamrata’s community center around 10:30 AM, the usual start of our workday. As usual, the team and I caught up while drinking hot *chai*. The daily team briefing had already started, when Nusrat, a community worker in her late 30s, arrived at the center. Dinesh, the team supervisor, reproached her for arriving late. Nusrat apologized, but her demeanor was calm and cheerful. As she took her place along the large circle in which we were all seated on the floor, she said she was late because there was a delay in the water supply that morning. “Usually water (flow) stops at 9:30,” she explained. Besides, she was also making arrangements for Ramzan Eid, which was only a few days away at the time. At one point she said, in her usual cheerful and calm disposition, “Don’t we (women) also have work (*kaam*) left at home?” And, in a move to placate Dinesh’s slight reprimand, she mentioned how she mobilized some basti women for that afternoon’s session along the way—a form of multitasking that other community workers, many of whom also lived in nearby bastis, engaged in.

As Nusrat settled in, the meeting resumed. Dinesh brought up the issue of assigning smartphones to the sakhis as part of Vinamrata’s efforts of using technology to map and monitor instances of domestic violence. However, he noted that despite giving smartphones to over a 100 women, there was not a commensurate rise in the number of expected case registrations—that is, when the sakhis identify survivors of abuse and violence and inform the community workers and counsellors. Dinesh said, “If sakhis aren’t being active … if they are not being vigilant, you (community team) have to work (harder) on cases!”

A few community workers responded that many sakhis informed them of problems over using smartphones, and that many did not understand their role within this restructured intervention. Bhavana, an experienced community worker and team supervisor, said that even though many sakhis were not filling the form on the smartphone application, cases of violence were still being reported offline. She said, “We need to take action (about this).” Dinesh added that supervisors need to stay abreast about the changes that occur with the smartphone application. We ate lunch around noon, after which each community worker proceeded to their respective areas to conduct sessions with sakhis and other women.

That afternoon, I joined Kalpana and Nazreen for the group activity they were about to facilitate. Kalpana was a team supervisor, and Nazreen was a community worker who had recently joined the team. By the time we reached the neighborhood, it had started raining heavily. We took shelter at a large open-air community space that had a high overhead roof. Nazreen was supposed to facilitate a session with a relatively new women’s group in the *chawl* (working class neighborhood), but when a few residents heard we were from Vinamrata they refused to let us use the space (and we could not have the session in the open due to the rains, either).

One senior resident, a retired army man, was especially vocal in his opposition. He cited a previous instance where Vinamrata’s health and nutrition program—a different “vertical” at the organization—had held an event in this very space but had invited Muslim women from nearby *jhopad-pattis* (slums), and had distributed food to them without giving any to the local residents. Kalpana was at pains explaining the difference between the programs, but the residents did not relent, and we had to eventually cancel the session.

### Fragmented Interventions and Vicarious Harm

As I noted above, sakhis, community workers, and crisis counsellors are part of the interlinked ecology of services and support that Vinamrata offers to survivors of violence. After sakhis or community workers identify and refer survivors to the counseling center, it is counsellors who work closely with them, providing psychosocial, therapeutic and legal support, particularly when working alongside institutions like courts. In crucial regards, counsellors too share and embody the critical consciousness of their community counterparts; many of them are trained in social work, and usually start their careers with grassroots work. I had the chance to interview some counsellors at the program during fieldwork in 2015, where our conversations covered their motivations, personal and professional trajectories, and experiences with the legal system.

The counsellors—who worked in offices located at different urban poor communities across the city—had just finished a day-long training workshop. I sat with them across a large, round table located in the hallway, for a group interview. In our conversation, the counsellors conceptualized gendered violence as relational and structural. One of them said, violence was having “control over another’s life,” like placing someone in “custody,” and not letting them “live like a human being.” They also linked violence to power relations, social systems, and everyday norms and behaviors which normalized injustice and “socialize a woman to violence.” Similarly, one of the counsellors who worked with the court systems presented a complex picture of the legal landscape, acknowledging that laws “can hurt … women.” Some of the main challenges she mentioned included inaccessibility of legal resources for urban poor women, delays in the judicial system, lack of non-legal supportive structures, economic dependency on abusive partners, and burdening survivors with the onus of responsibility.

Frontline workers and counsellors share these affective and critical understandings of inequality and violence. But in everyday life of the intervention they come to be positioned differently, as frontline workers also face pressures to meet their monthly case targets—something we observed in the previous ethnographic account, as well. In countless morning meetings I observed during fieldwork, questions like “Why aren’t cases visible?” or “Why is (the) work not showing?” were commonplace. In one meeting, for instance, community workers spoke of “high pressure cases”—those which involve serious self-harm or risk to survivors—and how sometimes there are gaps between their work and that of counsellors. Common refrains they heard from community women included “We come and go (but nothing happens)!” At times counsellors were also unable to undertake home visits, often due to high volume of cases (an issue that eventually gets resolved with several coordination meetings).

“This makes me feel that there is something lacking or wanting in me,” one community worker had said, explaining her dissatisfaction with such pressures of quantification. Another community worker shared an instance of a case-sharing meeting where a sakhi had asked “If there’s less violence, isn’t it a good thing?” The community worker continued, “We don’t give people anything (benefits), so we have to compete with other NGOs (who give material benefits).” Despite this, everyday work in the community extracts as much energy, if not more. During one particular meeting, for instance, a community worker tried to lighten the mood by remarking how it now appeared that they would have to instigate quarrels among couples to meet their targets—commenting on the stereotype held by men towards women’s organizations. Despite this, experienced workers like Bhavana reasoned that even though they witnessed partial stories and that someone “would be unhappy,” their work had to continue.

### Interstitial Intimacies, Caring Labor, and Navigating NGOization: A Discussion

The two ethnographic accounts discussed in this section outline the work that Vinamrata’s community workers were engaged in. Although they share social and gendered locations similar with that of sakhis—as urban poor women—community workers are also part of the social sector workforce. This workforce is highly feminized and, over the last 2 decades, has steadily experienced increased pressures of professionalization, quantification and precarity (Merry 2016; Roy 2019). We saw this most clearly in Nusrat’s case, which is in fact a common, mundane and unremarkable occurrence for millions of urban poor working women. At the same time, when the *chawl* residents confronted Kalpana and Nazreen over what they perceived to be infractions by members of another program, their critique actually marked out differences between them and the “other women”—signified by their religion and status—thus effectively drawing social boundaries.

Like the previous section, these accounts also show the salience of domestic intimacies in professionalized frontline work, signifying how women’s socially reproductive care transcends public–domestic dichotomies and ties together with neighborly socialities. As Snell-Rood (2015) shows in her ethnography with poor women in a Delhi slum, such socialities often serve as forms of “informal support” for survivors of violence. Frontline workers, too, are imbricated in such regimes of informal support; indeed, many of them are involved in providing care and support to survivors prior to becoming frontline workers (Chakraborty et al. 2017; Chakraborty 2021a). In this context, interstitial intimacies materialize through everyday rhythms of frontline work, where women like Nusrat navigate their domestic responsibilities whilst also meeting professional obligations, like mobilizing women and fostering collectives like women’s groups.

Still, these neighborly socialities are not a priori; as we saw in the *chawl* where Kalpana and Nazreen faced opposition, neighborly intimacies can often be fractured along lines of religion and community. The residents’ objections were inflected with both, majoritarian anxieties and biases (in opposing “Muslim slum dwellers”), and urban socialities and political networks, such as patronage or reciprocity (“We did not receive any benefits”) (see, respectively, Chatterji and Mehta (2007) and de Wit and Berner (2009)). Here, inasmuch as NGO interventions play an important role in creating solidarities, their status as outside actors—especially those that can provide benefits (*suvidha*)—problematize the very intimacies they facilitate in the first place.

Challenges to engendering interstitial intimacies also emanate from within organizational structures of NGOs, as we saw in both ethnographic accounts, though more explicitly in the second one. Even though community workers, sakhis and counsellors are part of a largely integrated and holistic response, such interventions come to diverge along organizational hierarchies. These observations raise important questions regarding the efficacy and legitimacy of NGOized interventions. How can we understand the scale and depth of the problem of violence against women when those entrusted with caring for and supporting survivors face such pressures? How do such pressures coexist with—or constrain and contract—the arduous, painstaking emotional and affective labor of building relationships with women?

Anthropologists writing on global paradigms of neoliberalism, quantification and NGOization, particularly in the domain of gender violence, focus their critiques on the following fronts. First, as Sally Engle Merry (2016) has shown, the proliferation of data-generation and quantification have resulted from universalization of norms and standards of measurement from the Global North and their circulation in the rest of the world as standardized indicators. Second, as Sharma (2006) and Mindry (2001) argue, the epistemic politics and organizational hierarchies of women’s NGOs—marked with the use of buzzwords like “development” or “empowerment”—are often premised on ethnocentric and imperial categories. Such terms and practices define beneficiaries as marginalized, feminized, oppressed and needing (western) development and aid. Third, as Roy (2019) shows in her recent ethnography of empowerment workers, NGOization, development and empowerment are implemented through disciplinary regimes of professionalization. Finally, as Haldane (2017) notes, such pressures often lead to decoupling of “agency care,” which is provided by individuals, from “structural care,” which refers to support provided to carers by institutions.

Conversations and dialogue with the team showed that such pressures were deeply felt in gender justice activism in India, especially after the neoliberal reforms of the Indian state in the 1990s. As political anthropologists like Ferguson (1996) and Gupta (2012) have observed, one of the unintended consequences of development discourse is the entrenchment of state power and burgeoning forms of inequity, even under neoliberal deregulation of healthcare and the social sector (Rao 2010). Feminist anthropologists, however, advocate for more nuanced and grounded explanations.

Writing on the theme of NGOization, for instance, Roy (2011, 590) suggests that we need to “argue against purist and dichotomized understandings of feminist activism and identities, and move, instead, towards points of convergence and hybridity.” In her ethnographic work with a large government organized NGO, Mahila Samakhya, Sharma (2006, 70–71) shows how these NGO workers wear “two hats”—using the vocabulary of the state to negotiate *authority*, and the rhetoric of the NGO, or social organization, to negotiate *legitimacy* in the rural communities. Similarly, Bernal and Grewal (2014b, 11, 14) recognize the adverse effects neoliberalism has had on feminist movements. Yet, NGO interventions continue to proliferate through what they call the “NGO form” which has become “a well-established element of the political landscape that itself is shaping the conditions of feminist struggles,” observing how changing relations between NGOs, states, and neoliberalism “produce changing feminist and female subjects.”

Within the wider landscape of neoliberalism and NGOization outlined so far, the import of interstitial intimacy lies in the fact that it engenders, and is engendered through, care. Frontline workers’ care and support to survivors of violence is a deeply intimate form of “caring labor,” a term I borrow from Susan Himmelweit (1999). Caring labor takes place in relationships where there is mutuality between carers and those they care for. As Himmelweit argues, the motivation “to care” and the reciprocity or recognition in “being cared for” is crucial in such affective encounters. Such skills are difficult to codify, and are “picked up in the course of developing a particular caring relationship” (34). Furthermore, Himmelweit’s use of caring labor draws on the concept of emotional labor or emotional work, a term coined by Arlie Hochschild (1983), which she defined as “the management of feeling to create a publicly observable facial and bodily display.” Emotional labor, Hochschild further writes, draws “on a source of self that we honor as deep and integral to our individuality” (for a wider discussion on care, see Tronto 1993; Mol 2008; Puig de la Bellacasa 2017; Mahadevan 2014).

Indeed, the centrality of care in frontline workers’ interventions keeps open possibilities of affective and ethical engagement. In a focus-group discussion I held with the team toward the end of fieldwork in 2015, Dinesh, who was also an experienced social activist, explained that as a result of neoliberalism many women’s organizations were accused of being “communist.” Turning a critical lens on organizational practices he was embedded in himself, he acknowledged that NGOs were able to benefit many people owing to structural limitations of the state—which was exemplary in urban poor neighborhoods, as such organizations and collectives maintained harmony after the 1992–93 anti-Muslim riots (Chatterji and Mehta 2007) and resisted dispossession and displacement of redevelopment projects (Weinstein 2014; Björkman 2020). Despite this, he noted, more and more organizations sought to implement projects or deliver material benefits to populations rather than engage in sustained movements.

The depoliticizing effects of NGOization and neoliberalism, ironically, can work toward making frontline work and gendered logics of care possible in the first place. Indeed, the language of projects can also be framed in gendered metaphors. For instance, across several conversations I have had with Bhavana and Kalpana, they observed that the very form and structure of NGOs meant that they were viewed as relatively neutral providers of services (and were thus often subject to claims of entitlements). Similarly, even though the segmented structure of interventions produced divergences between frontline workers and counsellors, community workers like Bhavana believe that such structures nevertheless enable certain forms of surveillance and vigilance. This was important so as to not let women’s groups go astray and work for the harm of the communities—which happened a few times in the past when former sakhis had started “settling” cases for sums of money (Chakraborty 2021b).

This illustrates a deep and reflexive understanding that frontline workers have toward the contexts they are a part of. But they also highlight the excesses of the same, for instance, which prevent them from operating (in) these contradictions, or undercut the metaphors and shorthands they use in everyday interventions. Kalpana, for instance, frames her NGOized work as beyond or ahead of electoral politics. Accordingly, if she “joins politics,” she will “have only one way (to work).” In contrast, she sees her present work as working “with everyone” (*sabke saath*). But she also recognizes that “Vinamrata won’t be there forever,” so building alliances with political classes and accruing social capital through NGOs is crucial, since it becomes a means towards an end, which always remains “working together with and helping people” (*logon ke saath mein madad karna*).

## Conclusion

In this article, I have drawn on ethnographic fieldwork with frontline workers involved in an NGOized program to prevent violence against women and girls to highlight convergences and entanglements between postfeminist practices and the NGOization of feminism and women’s movements. While the scholarship on postfeminism remains varied and contested, feminist interventions generally suggest that rather than marking a total departure from past feminist discourses and practices, postfeminism indexes complex—and at times contradictory—movements that assert particular forms of gendered and feminine subjecthood. For instance, such claims assert women’s agency within the contexts (and confines) of heterosexual relations, and the expression of expressedly feminine virtues and affectations.

I have used the heuristic interstitial intimacy to illustrate how the NGOization of feminist social work is a particular form of postfeminist practice that mobilizes frontline workers’ multiple, refracted and socially-inscribed and feminized identities. Interstitial intimacies enfold their domestic, affective and embodied intimacies with their neighborly, activist and professional engagements that are predicated on fostering collectives through intimate encounters, like training workshops and supporting survivors of violence. However, such intimacies are also affected by violence and marginalization—which not only necessitates interventions but also poses challenges to frontline work. NGOized work, with its pressures of professionalization and quantification, also constrains the ways that such intimacies engender open-ended and affective collectives.

Yet, as the ethnographic accounts presented in this article have shown, and as the convergences and entanglements between postfeminism and NGOization also demonstrate, frontline workers tend to appraise such overlapping and malleable ties between femininity and feminism (Mahadevan 2014) as crucial to their individual and collective subjectivation in mobilizations against violence. Even as they experience marginalization in material and structural terms, interstitial intimacies mark out emergent and relational forms of negotiation that are engendered in urban poor communities.

For instance, we see postfeminist inflections most clearly among the sakhis at Shivaji Nagar. Rather than articulate critical insights, like their peers in Dharavi, these sakhis asserted the importance of conjugality and domesticity. Yet, when we contextualize their articulations in wider social and biographical narratives and contexts, we see more nuanced logics emerge. The importance of conjugality and domesticity, for instance, have more complex histories in urban poor neighborhoods, relating as they do with the feminization of poverty and social and political marginalization (Roy 2003). The experiences of Dharavi sakhis, in contrast, drew on affective engagements with each other and with the organization. And although this workshop was generally more critical and reflexive, the sort of socialities they spoke of—forming connections, building relationships—bear a critical link with feminized logics seen in the Shivaji Nagar sakhis’ interventions: they were about care, specifically forms of care that were deeply related to the performance of emotional and affective labor, the disproportionate burden of which is borne by women (Chakraborty 2021a).

At the same time, sakhis’ and community workers’ close involvement with crisis counsellors embedded them within complex therapeutic regimes, as well as disciplinary practices of professionalization and quantification. Not only do these pressures lead to vicarious harm, but they profoundly unsettle the intimacies frontline workers share with community women and survivors of violence. Nevertheless, despite being part of a precarious workforce, frontline workers mobilize existing forms of socially reproductive care, wherein the at-times contradictory conjunction of care and professional social work lead to new forms of local practices. These practices have engendered unique forms of politics that exceed logics of neoliberal subjectivation, and reassert commitment to foundational feminist values and ethics, like care, relationality, and critique of patriarchal power.

## Data Availability

The datasets presented in this article are not readily available because the ethnographic data on which this article is based are sensitive in nature and cannot be made public. The data were obtained with informed oral consent and are presented anonymously/pseudonymized. Requests to access the datasets should be directed to proshant.chakraborty@gu.se.

## Footnotes

1 In addition to using pseudonyms for my research participants, I also use “Vinamrata” and “sakhi” to pseudonymize the NGO and their frontline workers. Although my collaborative research is available in the public domain, I have used pseudonyms as this article presents my anthropological interpretations.

2 With all but one instance—where I left a training session after a group of conservative, elderly Muslim women appeared rather inhibited with my presence—most women and sakhis found my presence rather unremarkable; and being present in such spaces and being viewed as part of Vinamrata also helped me interview sakhis for the study.

3 This activity was adapted from International Rescue Committee’s (2014) training manual, *Engaging Men in Accountable Practices*.

4 Such marginalization is particularly salient as the Shivaji Nagar sakhis’ experiences are part of the historical and social worlds of the M-East Ward, an area that has long been subject to political and economic marginalization, in large part due to its Muslim- and North India migrant-majority population (see, Björkman 2015; Björkman 2020). In contrast, the first ethnographic account had taken place Dharavi, which had a different historical trajectory. Even though Dharavi residents had contended with structural violence and communal violence, the neighborhood has long been home to various secular and rights-based social and political movements (Chatterji and Mehta 2007).
